# Principles of Effective and Robust Innate Immune Response to Viral Infections: A Multiplex Network Analysis

**DOI:** 10.3389/fimmu.2019.01736

**Published:** 2019-07-24

**Authors:** Yufan Huang, Huaiyu Dai, Ruian Ke

**Affiliations:** ^1^Department of Electrical and Computer Engineering, North Carolina State University, Raleigh, NC, United States; ^2^Department of Mathematics, North Carolina State University, Raleigh, NC, United States; ^3^T-6, Theoretical Biology and Biophysics, Los Alamos National Laboratory, Los Alamos, NM, United States

**Keywords:** immune response, interferon, viral infection, mathematical modeling, multiplex network

## Abstract

The human innate immune response, particularly the type-I interferon (IFN) response, is highly robust and effective first line of defense against virus invasion. IFN molecules are produced and secreted from infected cells upon virus infection and recognition. They then act as signaling/communication molecules to activate an antiviral response in neighboring cells so that those cells become refractory to infection. Previous experimental studies have identified the detailed molecular mechanisms for the IFN signaling and response. However, the principles underlying how host cells use IFN to communicate with each other to collectively and robustly halt an infection is not understood. Here we take a multiplex network modeling approach to provide a theoretical framework to identify key factors that determine the effectiveness of the IFN response against virus infection of a host. In this approach, we consider the virus spread among host cells and the interferon signaling to protect host cells as a competition process on a two-layer multiplex network. We focused on two types of network topology, i.e., the Erdős-Rényi (ER) network and the Geometric Random (GR) network, which represent the scenarios when infection of cells is mostly well mixed (e.g., in the blood) and when infection is spatially segregated (e.g., in tissues), respectively. We show that in general, the IFN response works effectively to stop viral infection when virus infection spreads spatially (a most likely scenario for initial virus infection of a host at the peripheral tissue). Importantly, we show that the effectiveness of the IFN response is robust against large variations in the distance of IFN diffusion as long as IFNs diffuse faster than viruses and they can effectively induce antiviral responses in susceptible host cells. This suggests that the effectiveness of the IFN response is insensitive to the specific arrangement of host cells in peripheral tissues. Thus, our work provides a quantitative explanation of why the IFN response can serve an effective and robust response in different tissue types to a wide range of viral infections of a host.

## Introduction

Virus infections and the resulting diseases are major challenges that our society faces today (1). One important determinant of the outcome of an infection is the innate immune response, particularly the type-I interferon (IFN) response (“the IFN response” for short). The IFN response is a highly optimized and general response that provides a critical first line of defense against a wide variety of virus infection (2). Failure to mount an effective IFN response against virus leads to systematic infection, while excessive IFN production leads to pathogenicity, severe symptoms or even fatality (2–4). It has been shown that the ability to evade host IFN response is an important determinant of viral replication (5–7), transmission (8), and host species range of viral infection (9). Viruses that lack the ability to evade the innate immune response are not able to infect and replicate in a host (7, 8). This demonstrates that the IFN response plays a crucial role in protecting hosts from virus invasion.

IFN molecules belong to a group of signaling proteins, known as cytokines, used by the immune system for cell-to-cell communication and induction of protective response. Upon infection, detection of viral RNA/DNA in the host cell triggers a signaling cascade and gene regulation, resulting in the production of IFNs (10). These IFNs then exit the infected cell and act as signaling molecules to bind to surface receptors located on the membranes of host cells (a process termed the IFN signaling), leading to induction of antiviral genes and thus an antiviral state in those cells (11). If an IFN molecule reaches an uninfected cell, i.e., paracrine signaling, this anti-viral state renders the cell refractory to viral infection. If an IFN molecule binds to the receptor of the infected cell that produces it, i.e., autocrine signaling, it inhibits viral replication and decreases the quantity of viral progeny being shed from that cell (6). Although the molecular mechanisms of the IFN response in individual cells have been well characterized (12), the collective dynamics of the host cell response arising from communications through IFN signaling and how the IFN response can effectively and robustly stop or suppress viral infections especially during the initial period of viral exposure in different peripheral tissues and different types of host cells are not understood.

To address these questions, we take a mathematical modeling approach using multiplex networks. Previous modeling works on virus dynamics and the IFN response focused on interpreting *in vitro* experiments and *in vivo* systematic infection dynamics (6, 13–17). For example, several elegant studies combining both single-cell experiments and mathematical modeling showed the importance of the timing of the IFN response in determining the outcome of an infection of a population of cells (6) and the importance of the IFN signaling in regulating the population response despite stochasticity in the single-cell level IFN response (16, 17). Two modeling works incorporated the IFN response into within-host viral dynamic models and showed that the IFN response can reduce the peak viral load during an influenza infection and explain the viral load plateau observed after peak viremia (13, 15). In this work, we introduce a multiplex network approach to understand virus invasion of a host and the immediate IFN response. In this framework, we assume in the multiplex network that virus and IFN molecules mediate contacts between cells through the infection layer and the protection layer, respectively. By considering different types of network topologies, i.e., reflecting host cell contact patterns, we show how the IFN response can effectively and robustly respond to virus infection especially in the initial site of viral exposure/infection where host cells are likely arranged spatially in the peripheral tissue.

## Methods

### The Multiplex Network Model Framework

**Figure 1:**
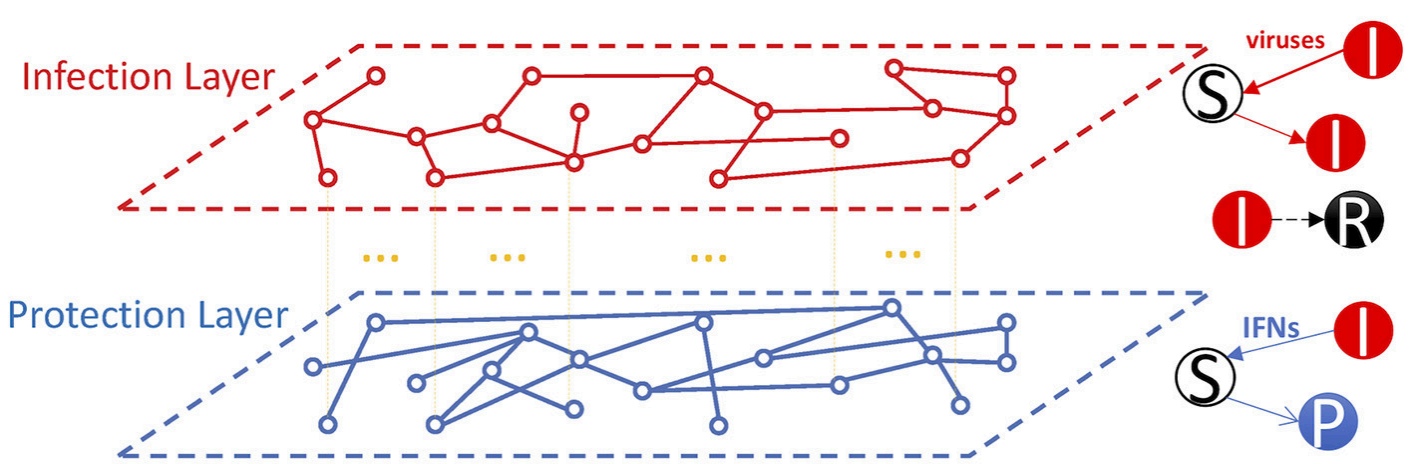
The multiplex network framework for the dynamics of virus infection and the IFN response. In the framework, cells are represented as nodes of the network. Two layers are considered, i.e., the infection layer and the protection layer. The nodes are shared between the two layers. Infected cells (I) produce viruses and IFNs (not considered explicitly in the model). Viruses infect susceptible cells (S) to become infected cells (I) through the infection layer, whereas IFNs spread and signal to susceptible cells to turn them to protected cells (P) through the protection layer. Infected cells die over time to become cells in the R class. We consider the impact of the overlap between the two layers and the topology of the two layers on the effectiveness of the IFN response to stop viral spread on the network.

In general, the multiplex network is modeled by a family of graphs ${\left\{G_{m}≜\;\left(V_{m},\;E_{m}\right)\right\}}_{m=1}^{M}$ where all graphs share the same set of nodes i.e., *V*_1_ = *V*_2_ = … = *V* = [*n*]. In our network models, we consider two layers of networks, i.e., the infection and the protection layers, and four types of cells, i.e., susceptible/target cells (S), infected cells (I), protected cells (P), and recovered/dead cells (R). The two layers share nodes (representing host cells) in the network; however, the two layers may have different edges that represent the infection or the protection of susceptible cells in the infection layer and the protection layer, respectively. The nodes have average degrees of ${\bar{k}}_{I}$ and ${\bar{k}}_{F}$ in the infection and the protection layer, respectively. Viruses and IFN molecules are not explicitly considered; instead, we assume that the contacts between infected cells and susceptible cells are mediated by viruses and IFNs through two layers in the network (Figure 1).

In this work, we consider two types of graphs for the two layers of a network. The first type is a well-mixed intralayer topology modeled by the Erdös-Rényi (ER) graph *G*(*n, p*) (18) in which a link exists between any two nodes with a uniform probability *p*. Then, the average degree of the ER graph is $\bar{k}=\left(n-1\right)p\approx \;np$. The second type is a spatial graph modeled by the 2-dimensional Geometric Random (GR) graph *G*(*n, r*) (19), in which a link exists between two nodes only when their 2-dimensional Euclidean distance is smaller than the prefixed range *r*, which we term the radius of diffusion. The radii of diffusion are *r*_*I*_ and *r*_*F*_ in the infection layer and the protection layer, respectively. The average degrees in the infection layer and the protection layer are calculated as $k_{I}=\left(n-1\right)\pi {r_{I}\;}^{2}\approx \;n\pi {r_{I}\;}^{2}$ and $k_{F}=\left(n-1\right)\pi {r_{F}\;}^{2}\approx \;n\pi {r_{F}\;}^{2}$, respectively. Simulation procedures of the network models are described in Huang et al. (20).

The following ordinary differential equations (ODEs) describe the mean field model of the infection and protection processes we consider in the networks:

$$\begin{array}{lll} \frac{dS}{dt} & = & -\beta SI-\;\varphi SI\\ \frac{dI}{dt} & = & \beta SI-\;\gamma I\\ \frac{dP}{dt} & = & \varphi SI\\ \frac{dR}{dt} & = & \;\gamma I \end{array}$$

In this model, susceptible cells (S) are infected at rate β or become protected at rate φ. Since we mainly focus on the initial infection dynamics, generation and death of susceptible cells are ignored. Infected cells (*I*) die at per capita rate γ to become cells in the *R* class. We assume that protected cells remain protected for simplicity, although anti-viral response in protected cells can be switched off over time (2, 15). Again, since we are mostly interested in the initial infection dynamics, ignoring the transition from protected cells to susceptible cells is a reasonable assumption. Here, we mainly focus on how the topology of a network impacts on the effectiveness of IFN to halt an infection through protecting susceptible cells, i.e., the paracrine IFN signaling. The impact of IFN on already-infected cells can be considered by extending the model with another infected class, i.e., infected cells that are at an antiviral state, and assume that infected cells in this class have a reduced viral production. However, this makes many analytical derivations impossible. Note that as a common practice in the network modeling approach, we rescale the four state variables against the total population size, such that *S* + *I* + *P* + *R* = 1. Then, *S, I, P*, and *R* in our network models represent the fraction of cells that are in their corresponding states.

### Analytical Derivations

To evaluate the impact of IFN on the infection threshold in the mean field/ODE model, we first define *R*_*I*_ as the reproductive number of the virus in the absence of IFN. We also refer this quantity as a measure of virus infectivity. It can be calculated as:

$$\begin{array}{l} R_{I}=\frac{\beta }{\gamma } \end{array}$$

We then define a quantity *R*_*F*_ for IFN similar as *R*_*I*_ for virus as:

$$\begin{array}{l} {\;R}_{F}=\frac{\varphi }{\gamma } \end{array}$$

Then, *R*_*F*_ is the average number of cells that an infected cell protects over its life time. Note that, protected cells do not further generate IFN and thus IFN signaling does not propagate in the absence of further infection. Thus, *R*_*F*_ is a single step measure of the effectiveness of the IFN signaling for individual cell response, and we refer this parameter as the individual-cell effectiveness of the IFN signaling.

The infection threshold β_*c*_ of the ODE model can be derived as: β_*c*_ = γ, i.e., as long as the infectivity parameter β is greater than the rate of recovery γ, the virus can cause sustained infection. Note the expression is independent of parameter φ, i.e., the parameter for the impact of IFN on protecting target cells. The infection threshold β_*c*_ of the network with two ER graphs and how it depends on the similarity between the two layers are derived previously in Huang et al. (20).

### Heterogeneity in the Susceptibility of Host Cells

To evaluate the impact of heterogeneity in the susceptibility of host cells, e.g., due to heterogenous receptor expressions, we assign each cell with a specific rate of infection, β, and this rate is drawn from a gamma distribution:

$$\begin{array}{l} \text{P}\left(\beta \right)=\frac{1}{\Gamma \left(k\right)\theta^{k}}\beta^{k-1}e^{-\frac{\beta }{\theta }} \end{array}$$

where *k* and θ are the shape and scale parameters, respectively, and Γ is the gamma function. In this way, the extent of heterogeneity is determined by the shape parameter *k*. The smaller *k*, the more heterogenous.

We follow the derivations in Huang et al. (20) to calculate the values of *R*_*I*_ for the simulations with heterogenous infection rate. First, we calculate the probability that a susceptible cell become infected when it is connected to an infected cell in the infection layer. Because infected cells die after a fixed period of time τ = 1 day in the simulation, this probability can be calculated as ζ = 1 − *e*^−βτ^ = 1 − *e*^β^, whose mean, $\bar{\zeta },$ is given by:

$$\begin{array}{l} \bar{\zeta }=\int_{0}^{\infty }\zeta P(\beta )d\text{β}=\int_{0}^{\infty }\left(1-e^{-\beta }\right)\frac{1}{\Gamma \left(k\right)\theta^{k}}\beta^{k-1}e^{-\frac{\beta }{\theta }}d\beta \\ \;=1-\frac{1}{{\left(1+\theta \right)}^{k}}. \end{array}$$

Then, the value of *R*_*I*_ is the product of $\bar{\zeta }$ and the average degree of the infection layer: $R_{I}={\bar{k}}_{I}\bar{\zeta }$.

## Results

### A Well-Mixed Model and a Network Model With Two Random (ER) Graphs

We first focused on multiplex networks where both layers are ER graphs as baseline models. In this framework, contacts between host cells (through viruses and IFNs) are random and there is no spatial structure in the contacts. These assumptions are reasonable for infections where cells move and contact with other cells (through viruses and IFNs) roughly randomly, for example, HIV infection in the blood. In our multiplex network model, the topologies of the graphs in the two layers, i.e., the contact structure between cells, can be explicitly modeled, in contrast to well-mixed models or single-layer network models. This allows us investigate how the IFN signaling through the protection layer competes with virus infection through the infection layer at the level of individual infected cells.

**Figure 2:**
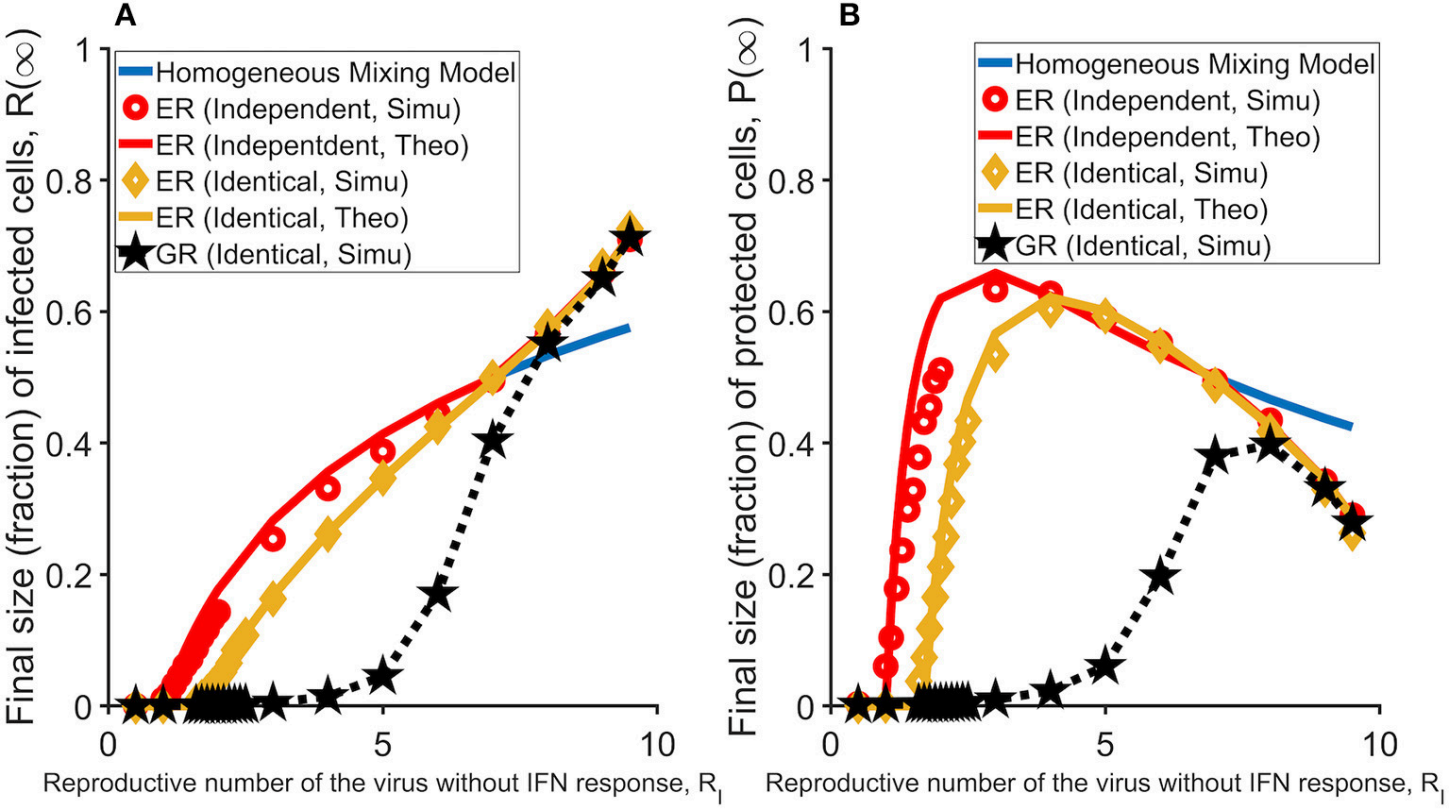
The effectiveness of the IFN response under different assumptions and topologies of the network. In general, protection of susceptible cells by IFN signaling, i.e., the IFN response considered in this study, works most effectively when viruses spread in a spatial manner (i.e., in the GR network). **(A)** The final sizes (fractions) of cells that are infected (and ultimately dead) at the end of the infection, *R*(∞), in the homogenous mixing model (blue line; partly overlaid by the red line) and the network models (in red, yellow, and black). Results are average of 1,000 simulations. **(B)** The final sizes (fractions) of cells that are protected at the end of infection, *P*(∞), in the homogenous mixing model (blue line) and the network models (in red, yellow and black). The individual-cell effectiveness of the IFN signaling, *R*_*F*_ is set to seven. The network model with two ER graphs (results of the model with two independent layers are in red; results of the model with two identical layers are in yellow), and the network model with two GR networks (in black). Lines denote analytical results derived in Huang et al. (20), whereas dots denote simulation results.

We considered two scenarios of the relationship between the two layers, i.e., the topologies of the two layers are independent of or identical to each other. We simulated the model and analyzed how the fractions of infected and then dead cells (a measure of the size of total infected cells) and protected cells (*R*(∞) and *P*(∞), respectively) changes with the infectivity of the virus (measured as *R*_*I*_; see Method). When the two layers are independent of each other, the subset of target cells that an IFN molecule can reach is independent from the subset that a virus (produced from the same cell as the IFN molecule) reaches, and thus there is no direct competition for target cells between viruses and IFNs at the individual infected cell level. We found that the predicted infection threshold value for virus infectivity, β_*c*_, i.e., the threshold value that viruses can cause sustained infection in a host, is independent of the parameter that governs the IFN protection of target cells, i.e., φ. On the other hand, when the two layers are identical (i.e., a more biologically relevant assumption), IFN molecules will reach to the same subset of target cells as the viruses produced from the same infected cell. In this case, the infection threshold becomes much larger than the threshold in the absence of IFN response, suggesting that IFN can prevent virus infection (the green line in Figure 2A). As we showed previously, IFNs inhibit viral spread effectively when IFNs reach the same subset of cells as viruses and thus reduce the number of susceptible cells that an infected cell can infect (20). Interestingly, these conclusions are similar to those in a previous network modeling work analyzing the impact of the spread of epidemic awareness on the transmission of infectious diseases (21). Further, we found that when viruses can cause infection, i.e., β > β_*c*_, there is a sharp increase in the number of protected cells (Figure 2B). This increase in protected cells prevents susceptible cells from being infected and thus the proportion of infected cells increases slowly with increases in *R*_*I*_ (Figure 2A).

### A Network Model With Two Spatial (GR) Graphs—IFN Can Effectively Halt Infection When Infection Is Spatial

For most viruses, initial viral infection events at the site of viral entry are expected to occur at the peripheral tissue where host cells are spatially structured. Spatial infection spread has also been shown to be a prominent infection mode of many viruses, especially for virus infections in the tissue (22–24). To evaluate the effectiveness of the IFN response in tissue, we constructed a multiplex network where the two layers are assumed to be GR graphs (see Methods). In the GR graph, we define nodes on a two-dimensional space and a maximal distance (i.e., radius of virus or IFN diffusion) such that an edge exists between two nodes only if the distance between two nodes is shorter than the radius of diffusion.

We simulated the model and found that strikingly, over a large parameter range of virus infectivity (measured by *R-*_*I*_), IFN protection of susceptible cells works much more effectively in the GR network than in the ER network. As shown in Figure 2A, the IFN response halts infection such that the total number of infected cells are kept at very low levels for a much wider range of virus infectivity. IFN protection also leads to a much lower total number of protected cells in the GR network than in the ER network (Figure 2B). This conclusion holds true as long as the individual-cell effectiveness of the IFN signaling (measured as *R*_*F*_; defined in Methods) is sufficiently high, e.g., when *R*_*F*_ > *R*_*I*_ (Figure S1).

**Figure 3:**
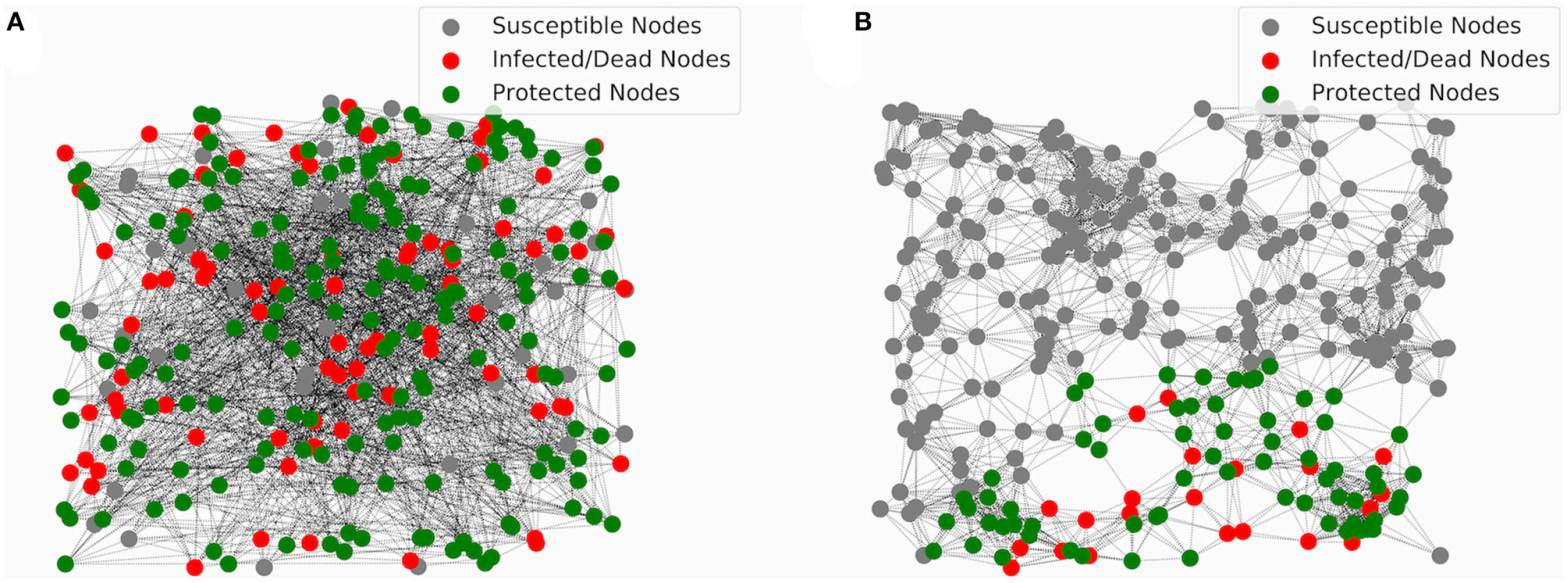
Visualization of simulations of the multiplex networks. **(A)** In the network with two ER graphs, most of the nodes are either infected and then dead (red) or protected (green) at the end of infection. **(B)** In the network with two GR graphs, most of the nodes are susceptible (gray) at the end of infection. Protection of host cells by the IFN signaling leads to an outer layer of protected cells that contain the infection at the local area of the initial infected nodes, i.e., the initial site of viral entry.

To understand why IFN protection of target cells works well in the GR network, we show two simulation realizations using networks assuming two ER graphs and two GR graphs in Figures 3A,B, respectively. In the network with two ER graphs (Figure 3A), connections/links between nodes are random. As a result, infection can propagate until most cells are either protected or infected/recovered. In contrast, cells in the GR network are connected only to neighboring cells in space. If the IFN response is strong enough, the IFN signaling can build up an outer layer of protected cells which effectively contains the infection near the site of initial infection. As a result, most of the cells (outside of the area of infection) stay susceptible without being infected (Figure 3B). Overall, the results suggest that the IFN response, i.e., the IFN signaling to protect susceptible cells, works extremely effectively when the virus spread spatially, a likely scenario for infections in tissues.

### Robustness of the IFN Response to Virus Infection in Tissue

The IFN response is a general response strategy employed by different types of host cells to prevent or suppress infections of a variety of viruses. This suggests that the IFN response works efficiently and robustly in a wide range of host cell or tissue environments. Here, we evaluated the robustness of the IFN response against variations in two assumptions in our model to understand how this collective host cell response work effectively despite heterogenous host environments.

**Figure 4:**
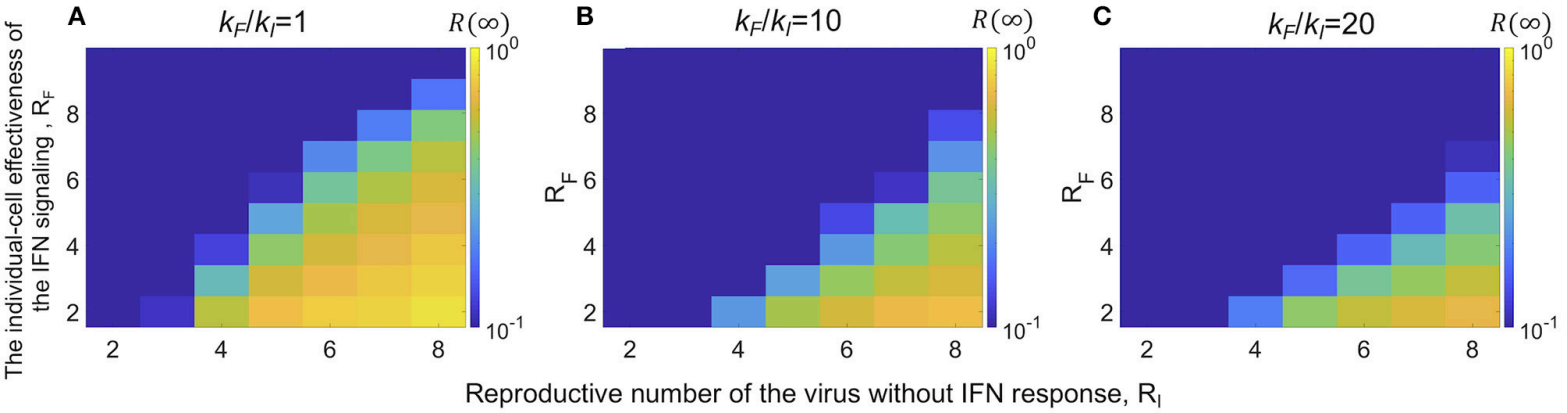
The effectiveness of the collective host cell IFN response is robust against variations in the ratio of the IFN diffusion over the virus diffusion in networks with two GR graphs. **(A)** The final sizes (fractions) of cells that are infected (and ultimately dead) at the end of the infection, *R*(∞) (color) for different virus infectivity (measured by *R*_*I*_) and the individual-cell effectiveness of the IFN signaling, *R*_*F*_. The ratio of the average degree in the protection layer over the average degree in the infection layer, *k*_*F*_/*k*_*I*_ is one. **(B,C)** The same plots as panel A except that *k*_*F*_/*k*_*I*_ = 10 and 20, respectively. In general, across the different ratios of *k*_*F*_/*k*_*I*_, the IFN response effectively suppresses virus spread (low *R*(∞) values; blue areas in the plots) as long as *R*_*F*_ > *R*_*I*_.

We first focused on one particular parameter that relates to the host tissue environment in our model: the diffusion coefficient of viruses and IFNs, i.e., the radius of the cell-cell edges (contacts) in the GR network. Due to differences in the viscosity of the fluid in the tissue and the layout of target cells, the ratio of the IFN diffusion over the virus diffusion and thus the ratio of the numbers of target cells they reach may differ in different tissue compartments. Below, we evaluate how the effectiveness of the overall IFN response changes with changes in these ratios. In the analysis, we varied the radius of the IFN diffusion in the protection layer (*r*_*F*_; defined in Methods), and assumed that the individual-cell effectiveness of the IFN signaling, *R*_*F*_, is constant. In this way, when the radius of IFN diffusion increases, the average degree of nodes in the protection layer (*k*_*F*_) increases; however, the protection rate per contact decreases. We explored how the final fraction of infection *R*(∞) changes with the ratio of the radius of IFN diffusion over the radius of virus diffusion, *r*_*F*_*/r*_*I*_. We found that there exists an optimal ratio, such that the total fraction of infection is minimized (Figure S2). Although the exact optimal ratio is parameter dependent, generally it occurs when the ratio is >1, i.e., the radius of IFN diffusion is similar or larger than the radius of virus infection. In general, when *R*_*F*_ > *R*_*I*_, there exists a wide range of ratios of IFN diffusion over virus diffusion that the IFN can suppress the virus infection below a very small fraction (blue areas in Figure 4). This suggests that as long as the IFN response is effective and diffuses similarly or faster than viruses, the IFN response is in general robust against variations in the IFN diffusion.

**Figure 5:**
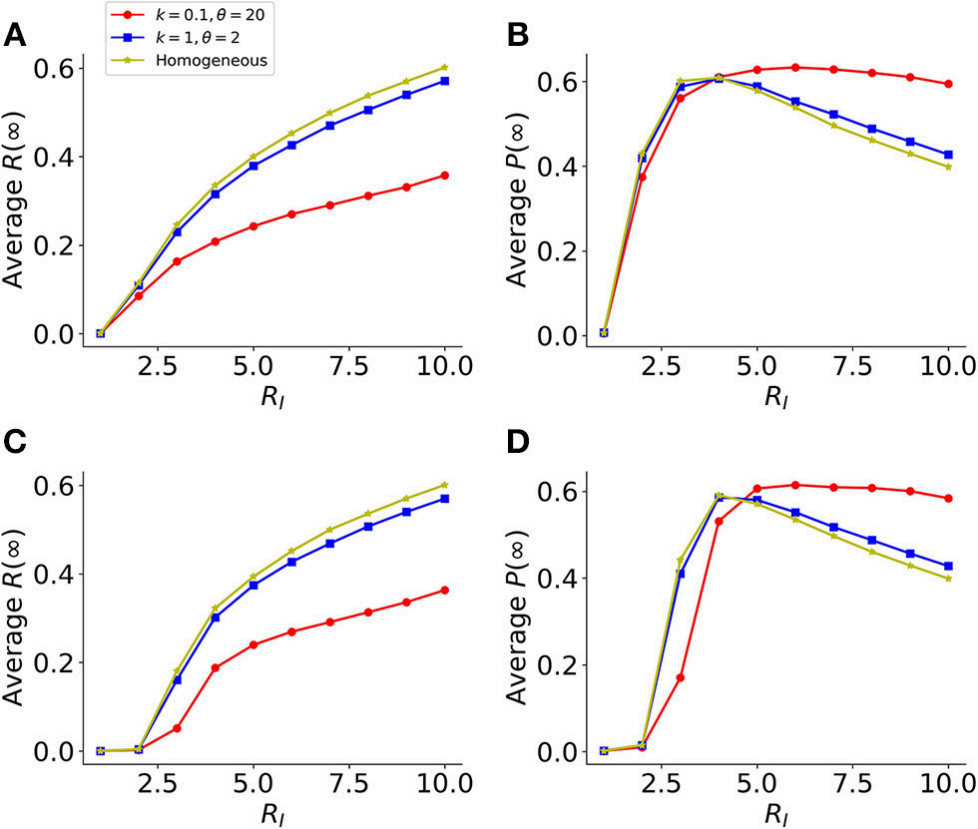
Heterogeneity in host cell susceptibility reduces the total size of infected cells. **(A)** The final sizes (fractions) of infected cells at the end of infection [Average *R*(∞)], in a model using two identical layers of ER graphs. Results are average of 1,000 simulations. Colored lines show simulations assuming different levels of heterogeneities in host-cell susceptibility. The heterogeneity is characterized by a gamma distribution with the shape parameter *k* and scale parameters θ. Note, the lower the value of *k*, the more heterogeneous the host cell susceptibility. **(B)** The corresponding final sizes (fractions) of protected cells at the end of infection, *P*(∞), in simulations shown in **(A)**. **(C,D)** Similar plots as in **(A,B)**, respectively, except that the model assumes GR graphs in the network. The individual-cell effectiveness of the IFN signaling, *R*_*F*_ is set to seven. The average degree of the networks is set to 40, such that the value of *R*_*I*_ reaches 10 in simulations assuming *k* = 0.1 and θ = 20.

In the analysis above, we assumed in the model that the host cells are a homogenous population of cells; whereas in reality, viruses typically infect a wide range of host cells and the host cells likely exhibit widely different levels of susceptibility to infection, e.g., as a result of heterogenous expression of receptors for viral infection (25–28). To evaluate the consequences of heterogenous host cell susceptibility to infection, we modified our model simulation to assume that each cell has a susceptibility drawn from a gamma distribution (instead of being the same), while keeping the rate of protection by IFNs, φ, constant (see Methods). The simulation results using ER and GR networks show that in general, the more heterogenous the host cell susceptibility (i.e., lower *k* values), the lower the final fraction of infection *R*(∞) (Figures 5A,C). This is because when host cell susceptibility is extremely heterogenous (e.g., the shape parameter *k* = 0.1 in the gamma distribution in Figure 5), the infection is driven a small fraction of highly susceptible cells. For the remaining large fraction of cells, they are much less likely to be infected than protected. Overall, this leads to a small fraction of cells being infected, yet the fraction of protected cells *P*(∞) remains similar across simulations (Figure 5). Therefore, the IFN response is effective to suppress viral infection when the susceptibility of host cells is heterogenous.

## Discussions and Conclusions

Here, we use a multiplex network approach to show how the collective host cell IFN response can effectively and robustly halt/suppress virus spread especially when viruses spread spatially. For a wide variety of viral infections, including influenza infection (22), HIV infection (29), mosquito borne viral infection, such as dengue (30) and zika (31), the site of entry is at the epithelium where target cells for infection are spatially arranged. The spread of viruses is thus expected to be a spatial process, i.e., infected cells only further infect a finite number of neighboring cells. We found that in this case, IFNs diffuses and signal to susceptible cells further away from infection, which builds up an outer layer of protected cells to contain infection locally. We also found that the collective IFN response is highly effective and robust against variations in parameter values that represent heterogenous host environments. This we argue is a property that allows the IFN response to be a general response employed by different types of host cells in peripheral tissues to respond to a wide variety of viruses to prevent viral establishment and invasion of a host at the initial site of the infection.

During systematic infection, viral infection process can be spatial or non-spatial. For viruses like HIV, infection in the blood and in the lymph nodes occurs among host cells that move around and contact each other randomly, the infection process may be better modeled using a random (ER) network. We show that in this case, the critical parameter that determines the effectiveness of IFN protection of target cells is the similarity between the infection layer and the protection layer (20). The higher the similarity, the more effective the IFN response. The IFN response can halt/suppress infection by directly competing with viruses at each individual cell level such that the number of target cells that each infected cell can infect is reduced. For many other viruses, e.g., influenza virus (22, 24) and HCV (23, 32), spatial viral spread may be prevalent throughout the infection course, if not the only infection mode.

The findings of our study, especially that IFN response is effective when infection spreads in a spatial manner, are consistent with a wide range of *in vivo* and *in vitro* observations. For example, imaging of liver biopsy from patients chronically infected with hepatitis C virus (HCV) showed that HCV infected cells form clusters and that IFN stimulated genes are highly expressed in infected cells as well as the surrounding susceptible cells. This strongly suggests effective IFN response to constrain cell-to-cell spatial spread in the liver (23). In another study (33), to understand the evolutionary trade-off of viral suppression of the IFN response, Domingo-Calap et al. compared the spread of a wild-type strain of the vesicular stomatitis virus to a mutant strain that stimulates stronger IFN response than the wild-type. Real-time fluorescence microscopy showed that in contrast to a faster and homogenous spread of the wild-type virus in monolayer host cells, the mutant viruses spread slower and infected cells form clusters. This again suggests that the IFN response triggered by the mutant acts to constrain infection. Interestingly, when the monolayer spatial structure of host cells is disrupted, the mutant grew faster than the wild-type in well-mixed culture. This is consistent with the results we show in this study that spatial structure is a key determinant of the effectiveness of the IFN response. Overall, these experimental observations support our model predictions, and thus, our model serves a useful tool to understand the quantitative principles of the IFN response. These understandings may lead to development of effective therapies/vaccines to prevent virus transmission and infection (5–8).

Overall, our results suggest that considering the topology of the spreading process is critical to the understanding and prediction of the impact of collective IFN response arising from host cells. Therefore, experimental studies that examine the contact structure and topology for an infection process would help to parameterize the model to make precise predictions. Here our work considered two distinct scenarios of the topology of the spreading process, i.e., the random (ER) network and the spatial (GR) network. An actual infection *in vivo* may involve both spatial and non-spatial contacts. For example, it has been shown HCV mostly spread to neighboring cells, forming clusters of infected hepatocytes in the liver; while it is also able to have a long-range dispersal to hepatocytes through blood flow (23, 32). Similar patterns of foci of infection are also observed for influenza virus (22). Further work is warranted to consider network structures that incorporate both spatial spread and random spread, and evaluate the effectiveness of IFN response in those settings.

Given that the IFN response is a highly optimized and highly effective general response against viruses (2), we argue that the strategies employed by IFN and the results derived from this work could shed light on or lead to solutions to problems in other disciplines. For example, network models are frequently used in the modeling of epidemics to understand how infection dynamics or control strategies are impacted by network topologies (34–36). Furthermore, we speculate that the understanding of the population IFN response may lead to bio-inspired strategies for controlling rumor spreading in social networks or cyberattacks in computer networks.

## Author Contributions

YH and RK derived and analyzed the theoretical models. YH conducted the computer simulations. All authors analyzed the data, wrote and edited the manuscript and contributed to the development of ideas.

### Conflict of Interest Statement

The authors declare that the research was conducted in the absence of any commercial or financial relationships that could be construed as a potential conflict of interest.
