# Supplementary material for: Principles of Effective and Robust Innate Immune Response to Viral Infections: A Multiplex Network Analysis
Source: Front Immunol. 2019 Jul 24;10:1736. doi: 10.3389/fimmu.2019.01736 (PMC6667926; doi:10.3389/fimmu.2019.01736)
Supplement: Supplementary file 1 [file Data_Sheet_1.PDF]

Supplementary Material

Principles of robust innate immune response to viral infections: a multiplex network analysis

Yufan Huang[1], Huaiyu Dai[1, *], Ruian Ke[2, 3*]

[1]Department of Electrical and Computer Engineering, North Carolina State University, Raleigh, North Carolina, USA

[2]Department of Mathematics, North Carolina State University, Raleigh, North Carolina, USA

[3]T-6, Theoretical Biology and Biophysics, Los Alamos National Laboratory, Los Alamos, USA

*** Correspondence:**
Corresponding Authors: R.K.: rke@lanl.gov; H.D.: hdai@ncsu.edu

1 Supplementary Figures

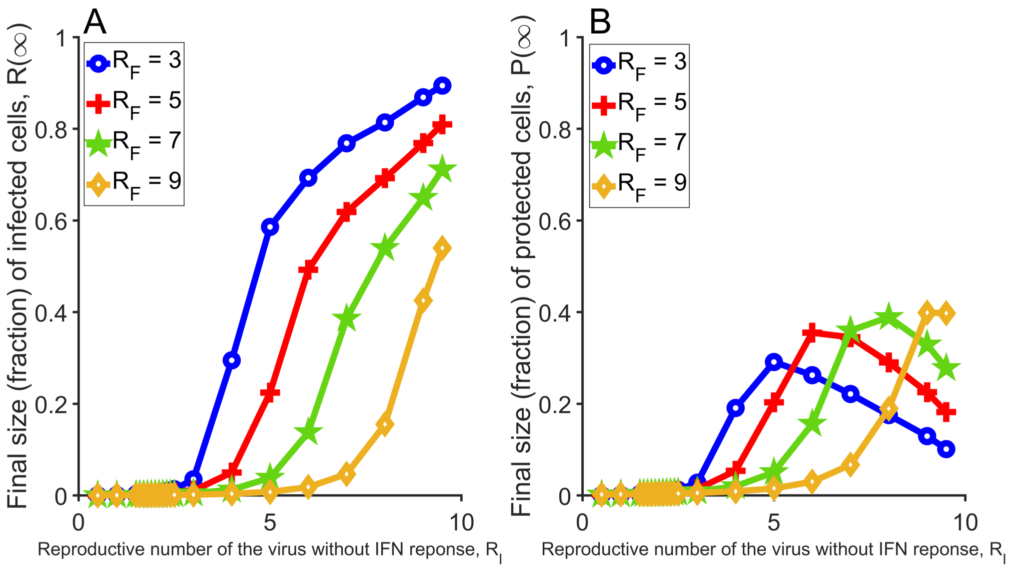

Figure S1. The IFN response effectively halt/suppresses virus spread in networks with two GR graphs when $R_F > R_I$. Similar simulation results as shown as the black line in Fig.2 in the main text, except that $R_F = 3$, 5, 7 and 9.

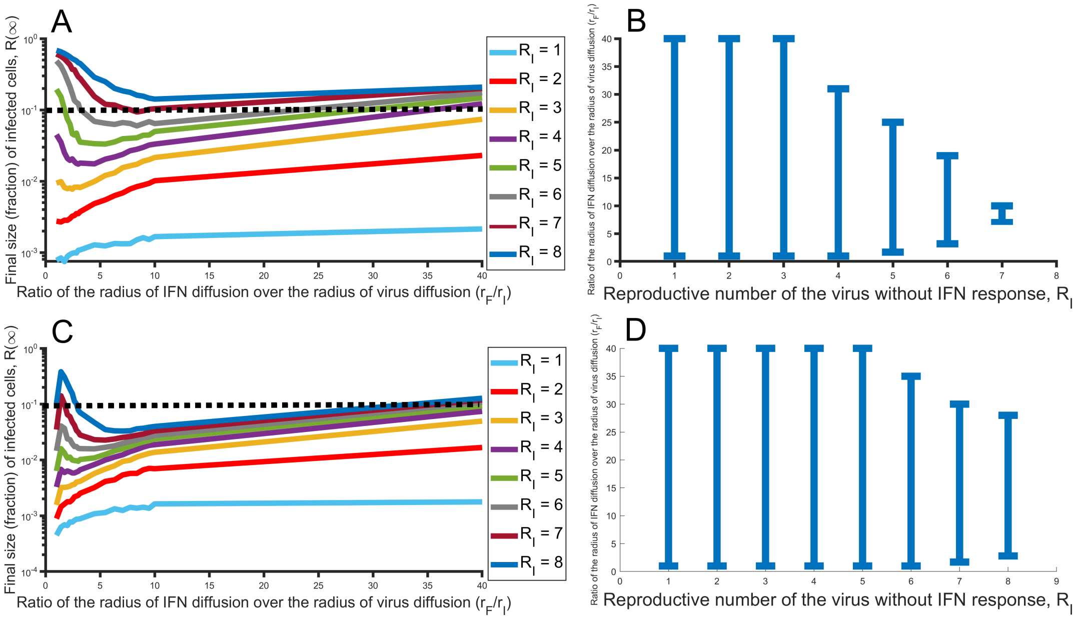

Figure S2. IFN response is robust against variations in the radius of IFN spread, and can effectively stop a viral infection across a large parameter space in the network with two GR graphs. (A) The final sizes (fractions) of cells that are infected (and ultimately dead) at the end of the infection ($R(\infty)$) for different radii of IFN spread relative virus spread. The interferon response can keep ($R(\infty)$) at a low level (< 0.1; dashed line) for a wide range of parameter values. R_F=5. **(B)** The ranges of ratio of the radius of IFN spread over the radius of virus spread (r_F/r_I) that lead to $R(\infty) < 0.1$ for different viral infectivities, R_I. i.e. the ranges of r_F/r_I values where $R(\infty) < 0.1$ in panel A. (C and D) similar plots as panels A and B, respectively, except that R_F=9.
